# Are you now a good surgeon? T2 positive margin status as a quality outcome measure following radical prostatectomy

**DOI:** 10.1007/s00345-016-1836-0

**Published:** 2016-04-25

**Authors:** Arees Damani, Mieke Van Hemelrijck, Wahyu Wulaningsih, Danielle Crawley, Declan Cahill

**Affiliations:** 1 Cancer Epidemiology Group, Division of Cancer Studies, Research Oncology, School of Medicine, Guy’s Hospital, King’s College London, 3rd Floor, Bermondsey Wing, London, SE1 9RT UK; 2 The Royal Marsden NHS Foundation Trust, London, UK

**Keywords:** Radical prostatectomy, Outcomes assessment, Prostate cancer, Positive margins, Laparoscopic surgery

## Abstract

**Objective:**

To assess potential biases, such as the reporting pathologist, that may affect objectivity of T2 positive margin rates as a quality outcome measure following radical prostatectomy.

**Patients and methods:**

Prospective data on 183 consecutive LRP patients with pT2 disease, operated on by a single surgeon (2003–2009), were studied. Outcomes were grouped as pre-, peri-, and post-operative and included: age, ethnicity, Gleason score, reporting pathologist, percentage of positive cores, operative time, blood loss, nerve-sparing status, hospital stay and prostate weight. Descriptive analysis and logistic regression analysis were carried out to compare these variables by positive margin status.

**Results:**

A total of 30 (16.4 %) positive surgical margins (PSMs) were reported. Surgical stage, earlier date of surgery, and lower prostatic weight showed statistically significant associations with PSM status in both univariate and multivariate analysis. The reporting pathologist was not found to be predictive of PSMs (*P* = 0.855).

**Conclusion:**

We showed that the reporting pathologist does not influence T2 positive margin status, in contrast to tumour characteristics and surgeon experience. T2 positive margin assessment therefore appears to be an objective quality outcome measure.

## Introduction

The gold standard measure of surgeon performance for radical prostatectomy would include patient reported outcomes such as continence, quality of life with PSA follow-ups; however, this is rarely practical in the clinical setting. There is therefore a need for a more easily attainable, unbiased marker of surgical performance. We recently showed that T2 positive margin rate is the most informative quality outcome measure with the least potential observer bias [1]. This highlights the possible use of T2 positive margin rates as a single objective outcome measure indicative of surgeon skill. Positive surgical margins (PSMs) have been studied in depth to analyse their clinical implications following radical prostatectomy (RP). Stephenson et al. conducted a study looking at 11,521 patients who underwent a RP between 1987 and 2005 and found no association between margin status and cancer-specific mortality within 15 years post-RP. However, they did observe a positive association with biochemical recurrence, so that it is recommended to avoid PSMs where possible [2].

Intra-prostatic incision (IPI) is associated with significantly increased biochemical recurrence (BCR), compared to patients with negative surgical margins with or without extra-prostatic extension. The 5-year biochemical recurrence-free survival was only 77 % for patients with intra-prostatic incision, compared to 94 % for those without (*P* ≤ 0.0001), according to Preston et al. [3]. Similar results have also been shown in other studies [4, 5]. There is thus a growing body of evidence to support PSMs as a prognostic factor indicative of biochemical recurrence following RP.

In addition to its value as a diagnostic predictor, PSM have been suggested to be a good indicator of surgeons’ performance. Vickers et al. [6] showed that surgeons who performed higher volumes of RP showed a decrease in PSMs over time, with a plateau after 250 procedures—suggesting that PSM rates can be used to monitor surgeons’ experience. Although this data were published for the open procedure, laparoscopic procedures have a similar learning curve as shown by Secin et al. [7] who showed a similar learning curve plateauing after 200–250 cases. Whilst pathological factors have been shown to influence margin status, surgeon skill and experience also may play an important role. Eastham et al. [8] showed that high-volume surgeons had significantly lower PSM rates. It has been suggested that intra-prostatic incision may be a better quality measure of surgeon experience than PSM rates [9]. However, rates of intra-prostatic incisions vary greatly between studies. This is potentially due to the difficulty in differentiating between true T2 positive margins and extra-prostatic extension [9–11].

We have therefore evaluated the clinical variables that may affect T2 PSMs to determine whether this can be used as an objective measurement of surgeon skill and experience.

## Methodology

### Study population

We obtained prospective data for 183 consecutive patients who underwent laparoscopic radical prostatectomy (LRP) between 2003 and 2009. A single surgeon carried out all procedures. The surgeon reported all data pre-, peri- and post-operatively. All data were also verified in the electronic medical records. Only patients with T2 stage disease as classified by TNM staging [12] were included in the study.

### Margin status

Information on margin status was collected post-operatively. A PSM was reported when cancer cells were seen touching or extending beyond the inked resection margin, and were all identified in one Pathology Department by 10 different consultant pathologists, using standard procedures. PSM location was described as apical, circumferential or both. The pathologists are all dedicated consultant uropathologists employed in a teaching hospital. Inter-pathologist review of specimens is carried out as part of an internal-audit.

### Clinical variables

Pre-operative, peri-operative, and post-operative variables were studied in relation to margin status.

Pre-operative variables included age at surgery, ethnicity, Gleason Score (primary and secondary), TNM staging, PSA, and percentage positive cores. Age at surgery was categorised into ≤60 and >60 as older age is an important clinical determinant of prostate cancer [13]. Total Gleason score was divided into three categories (≤6, 7, ≥8) [14], which corresponded to different survival profiles [15]. PSA was divided into categories of ≤10 and >10 ng/ml. The percentage of positive cores was studied as a categorical variable (<25 %, 25–49 %, ≥50 %). Date of surgery was studied as a categorical variable (2003–2004, 2005–2006, 2007–2009), as a measure of increasing surgeon experience.

Peri-operative variables were reported by the surgeon and included operative time, blood loss and neurovascular bundle (NVB) resection status. NVB status was assessed as no NVB, unilateral and bilateral NVB. Operative time (minutes) was measured as the time from the first incision to the last suture. Blood loss (ml) was recorded from the suction system in theatre.

Post-operative variables studied included prostate weight as reported by the pathologists. Length of hospital stay (days) obtained from the medical record.

### Statistical analysis

Statistical analysis was carried out using Statistical Analysis Systems (SAS) release 9.4 (SAS Institute, Cary, NC).

Univariable logistic regression analysis was performed for each clinical variable to assess their associations with margin status. A test for trend was conducted by assigning categories as an ordinal scale. To further assess this association while adjusting for potential confounding factors, multivariable analysis was performed by incorporating all clinical variables in the model. Finally, to assess any differences by margin location only in patients with PSM (*n* = 30), one-way ANOVA and Chi-square tests were performed for each clinical variable with respect to the three margin locations (apical, circumferential or both).

## Results

Descriptive statistics of the patient cohort for pre-operative variables are presented in Table 1, with peri- and post-operative variables in Table 2. 53 % of patients were >60 years of age at time of surgery. The majority was of a white background and presented with PSA ≤10 ng/ml (77 %), with a Gleason score of ≤6 (60 %). NVB resection was performed unilaterally in 29 patients (16 %) and bilaterally in 68 (37 %). Mean hospital stay was 2 days. 49.7 % of procedures were carried out between 2007 and 2009, when the surgeon was more experienced.

**Table 1:** Descriptive statistics for pre-operative variables

	Frequency/mean	%/SD
Pre-operative variables		
Age at surgery (years)		
≤60	86	47.3
>60	96	52.7
Date of surgery		
2003–2004	44	24.0
2005–2006	48	26.2
2007–2009	91	49.7
Ethnicity		
Black	37	20.2
White	134	73.2
Other	3	1.6
Missing	9	4.9
PSA (ng/ml)		
≤10	141	77.0
>10	40	21.9
Missing	2	1.3
Percentage cores positive (%)		
<25 %	55	30.1
25–49 %	56	30.1
≥50 %	38	20.8
Missing	34	18.6

**Table 2:** Descriptive statistics for peri, and post-operative variables

*Peri-operative variables*		
Mean blood loss (ml)	279	225
Mean operative time (min)	159	37
Nerve-sparing status		
None	38	20.8
Unilateral	29	15.8
Bilateral	68	37.2
Missing	48	26.2
*Post-operative variables*		
Mean hospital stay (days)	2	2.12
Prostate weight (g)		
20–59	100	54.6
60–99	49	26.8
≥100	13	7.1
Missing	21	11.5
Pathologist		
1	67	36.6
2	7	3.8
3	10	5.5
4	53	29
5	9	4.9
6	1	0.5
7	11	6.0
8	1	0.5
9	5	2.7
10	4	2.2
Missing	15	8.2
Surgical stage		
T2a	30	16.4
T2b	52	28.4
T2c	96	52.5
T2x	5	2.7
Total Gleason		
≤6	109	59.6
7	60	32.8
≥8	13	7.1

Surgical stage showed a statistically significant correlation with PSM status in both univariate (*P* = 0.035) (Table 3) and multivariate analysis (*P* = 0.004) (Table 4). Patients with higher surgical stage of T2b and T2c were more likely to have PSMs (OR 8.7 (95 % CI 1.07–70.71) and OR 4.1 (95 % CI 0.52–33.27), respectively) than those with T2a disease. Earlier date of surgery was statistically significantly associated with higher PSM rates in both univariate (Table 3) and multivariate analysis (Table 4) (*P* = 0.018). PSMs were also most prevalent in patients with prostatic weight between 20 and 59 g. PSMs were not associated with the reporting pathologist (*P* = 0.855).

**Table 3:** Univariate analysis of pre, peri and post-operative variables

	Positive surgical margin (*N* = 30)	Negative surgical margins (*N* = 153)	Odds ratio	95 % Confidence interval	*P*_trend_
*Pre-operative variables*					
Age at surgery (years)					0.057
≤60	19 (22 %)	67 (78 %)	1	Ref	
>60	11 (11 %)	85 (89 %)	0.46	0.20–1.02	
Date of surgery					0.011
2003–2004	13 (30 %)	31 (70 %)	1	Ref	
2005–2006	7 (15 %)	41 (85 %)	0.41	0.15–1.14	
2007–2009	10 (11 %)	81 (89 %)	0.29	0.12–0.74	
Ethnicity					0.052
Black	9	28	1	Ref	
White	21	113	0.58	0.24–1.40	
Other + missing	0	12	n/a	n/a	
PSA (ng/ml) (SD)					0.093
≤10	27	114	1	Ref	
>10	3	37	0.34	0.10–1.19	
Percentage cores positive (%)					0.720
<25 %	8 (15 %)	47 (85 %)	1	Ref	
25–49 %	10 (18 %)	46 (82 %)	1.28	0.46–3.52	
≥50 %	8 (21 %)	30 (79 %)	1.57	0.53–4.62	
Missing	4	30	0.78	0.22–2.83	
*Peri-operative variables*					
Mean blood loss (mls) (SD)	299.8 (363.7)	274.8 (185.4)	1.00	0.99–1.00	0.580
Mean operative time (min) (SD)	160.8 (39.06)	158.0 (37.1)	1.00	0.99–1.01	0.708
Nerve-sparing status					0.219
None	9	29	1	Ref	
Unilateral	4	25	0.52	0.14–1.88	
Bilateral	11	57	0.62	0.23–1.67	
Missing	6	42	0.46	0.23–3.47	
*Post-operative variables*					
Mean Hospital stay (days) (SD)	2.07 (1.2)	2.02 (2.26)	1.01	0.84–1.21	0.911
Prostate Weight (g)					0.035
20–59	24 (24 %)	76 (76 %)	1	Ref	
60–99	3 (5 %)	46 (95 %)	0.21	0.06–0.72	
≥100	2 (15 %)	11 (85 %)	0.58	0.12–2.78	
Missing	1	20	0.16	0.02–1.24	
Pathologist					0.855
1	10	57	1	Ref	
2	4	3	7.60	1.47–39.21	
3	1	9	0.63	0.07–5.56	
4	11	42	1.49	0.58–3.84	
5	0	9	n/a	n/a	
6	0	1	n/a	n/a	
7	3	8	2.14	0.48–9.46	
8	0	1	n/a	n/a	
9	1	4	1.43	0.14–14.10	
10	0	4	n/a	n/a	
Surgical stage (%)					0.035
T2a	1	29	1	Ref	
T2b	12	40	8.70	1.07–70.71	
T2c	12	84	4.14	0.52–33.27	
T2x	5	0	n/a	n/a	
Total Gleason					0.187
≤6	22	87	1	Ref	
7	6	54	0.44	0.17–1.15	
≥8	2	11	0.72	0.15–3.48

**Table 4:** Multivariate analysis of pre, peri, and post-operative variables

	Multivariable		
	OR	95 % CI	*P*_trend_
*Pre-operative variables*			
Age at surgery (years)			0.042
≤60	1	Ref	
>60	0.24	0.06-0.95	
Date of surgery			0.018
2003–2004	1	Ref	
2005–2006	0.44	0.04–5.22	
2007–2009	0.31	0.03–3.62	
Ethnicity			0.070
Black	1	Ref	
White	0.71	0.19–2.70	
Other + missing	n/a	n/a	n/a
PSA (ng/ml)			0.131
≤10	1	Ref	
>10	0.29	0.06–1.45	
Percentage cores positive (%)			0.898
<25 %	1	Ref	
25–49 %	1.57	0.32–7.62	
≥50 %	1.83	0.38–8.84	
Missing	0.97	0.13–7.19	
*Peri-operative variables*			
Blood loss (mls)	1.00	0.99–1.00	0.576
Op time (min)	0.99	0.97–1.01	0.458
Nerve-sparing status			0.037
None	1	Ref	
Unilateral	0.24	0.03–1.72	
Bilateral	0.19	0.04–1.00	
Missing	0.17	0.03–1.01	
*Post-operative variables*			
Surgical stage			0.004
T2a	1	Ref	
T2b	6.52	0.63–67.01	
T2c	7.52	0.50–113.26	
T2x	n/a	n/a	
Prostate weight (g)			0.031
20–59	1	Ref	
60–99	0.35	0.08–1.52	
≥100	1.29	0.13–12.96	
Missing	0.16	0.02–1.64	
Total Gleason			0.148
≤6	1	Ref	
7	0.18	0.04–0.90	
≥8	0.63	0.08–4.84	
Mean hospital stay (days)	0.88	0.63–1.21	0.427

In contrast with the univariate analysis, larger prostatic weight (>100 g) showed a higher predictive value for PSM in multivariate analysis (Table 4). Multivariable analysis also showed NVB resection status to be associated with PSMs, with more PSM in patients in whom NVB resection was not attempted (*P* = 0.037).

Finally, we assessed whether any of the clinical variables studied were differentially distributed across PSM locations (Table 5). Highest mean blood loss was observed in multifocal PSMs (apex and circumferential) (883.3 ml, *P* = 0.006). Patients with apical PSMs mostly had T2c disease, whereas those with circumferential PSMs had the highest proportion of T2b disease (*P* = 0.01).

**Table 5:** Analysis of variables by positive margin location

	Apex (%) *N* = 15	Circumferential (%) *N* = 12	Apex and circumferential (%) *N* = 3	*P* value
Age at time of surgery (years)				0.898
≤60	10 (62.5)	7 (58.3)	2 (66.7)	
>60	5 (31.3)	5 (41.7)	1 (33.3)	
Missing	1 (6.3)	0 (0.0)	0 (0.0)	
Ethnicity				0.924
Black	4 (26.7)	4 (33.3)	1 (33.3)	
White	11 (73.3)	8 (66.7)	2 (66.7)	
Other	0 (0)	0 (0)	0 (0)	
Mean PSA (ng/ml)				0.189
≤10	12 (80.0)	12 (100)	3 (100)	
>10	3 (20.0)	0 (0)	0 (0)	
Mean percentage cores positive (%)				0.057
<25 %	0 (0)	7 (58.3)	1 (33.3)	
25–49 %	7 (46.7)	2 (16.7)	1 (33.3)	
>50 %	5 (33.3)	2 (16.7)	1 (33.3)	
Missing	3 (20.0)	1 (8.3)	0 (0)	
Mean blood loss (mls) (SD)	182.33 (87.7)	300.83 (237.2)	883.33 (970.0)	0.006
Mean operative time (min) (SD)	153 (34.4)	170.83 (45.9)	160 (34.6)	0.515
Mean hospital stay (days) (SD)	2.27 (1.3)	1.67 (0.7)	2.67 (2.1)	0.297
Mean prostate weight (g)				0.442
20–59	12 (80.0)	10 (83.3)	2 (66.7)	
60–99	2 (13.3)	1 (8.3)	0 (0.0)	
≥100	0 (0.0)	1 (8.3)	1 (33.3)	
Missing	1 (6.7)	0 (0.0)	0 (0.0)	
Pathologist				0.144
1	7 (30.4)	3 (15.8)	0 (0.0)	
2	1 (4.3)	2 (10.5)	1 (33.3)	
3	0 (0.0)	1 (5.3)	0 (0.0)	
4	5 (21.7)	5 (26.3)	1 (33.3)	
5	0 (0.0)	0 (0.0)	0 (0.0)	
6	0 (0.0)	0 (0.0)	0 (0.0)	
7	2 (8.7)	1 (5.3)	0 (0.0)	
8	0 (0.0)	0 (0.0)	0 (0.0)	
9	0 (0.0)	0 (0.0)	1 (33.3)	
10	0 (0.0)	0 (0.0)	0 (0.0)	
Missing = 15	8 (34.8)	7 (36.8)	0 (0.0)	
Nerve-sparing status				0.535
None	3 (20.0)	5 (41.7)	1 (33.3)	
Unilateral	2 (13.3)	1 (8.3)	1 (33.3)	
Bilateral	5 (33.3)	5 (41.7)	1 (33.3)	
Missing	5 (33.3)	1 (8.3)	0 (0.0)	
Surgical stage				0.010
T2a	0 (0.0)	0 (0.0)	1 (33.3)	
T2b	4 (26.7)	8 (66.7)	0 (0.0)	
T2c	9 (60.0)	2 (16.7)	1 (33.3)	
T2x	2 (13.3)	2 (16.7)	1 (33.3)	
Total Gleason				0.626
″6	10 (62.5)	10 (83.3)	2 (66.7)	
7	3 (18.8)	2 (16.7)	1 (33.3)	
≥8	2 (12.5)	0 (0.0)	0 (0.0)	
Missing	1 (6.3)	0 (0.0)	0 (0.0)	
Date of surgery				0.751
2003–2004	5 (33.3)	7 (58.3)	1 (33.3)	
2005–2006	4 (26.7)	2 (16.7)	1 (33.3)	
2007–2009	6 (40.0)	3 (25.0)	1 (33.3)	

## Discussion

Our multivariate analyses showed that several clinical variables are predictive of T2 PSMs, including nerve-sparing status and tumour characteristics. Fewer PSM were seen with later date of surgery, confirming the notion of a surgical learning curve in RP procedures. The reporting pathologist and Gleason score were not associated with T2 positive margin status.

PSMs have been extensively studied to determine their clinical significance. Several studies have outlined the prognostic value of PSMs [2, 10, 16–20]. It is clear that while PSMs are not strongly linked to cancer-specific survival, PSMs are an important predictor of BCR. For these reasons, PSM rates are increasingly being reported as a quality measure, indicating superior surgeon experience and skill. However, few studies to date have investigated factors that may influence margin status apart from surgical experience and tumour characteristics. Although we limited our analysis to patients with pT2 stage, we assessed several clinical variables predictive of prostate cancer prognosis including, pre-operative PSA and Gleason score.

Surgeon experience has a large impact on PSM rate. Vickers et al. [21] showed that PSM rates were significantly improved when treated by a surgeon who had completed at least 250 procedures compared to a surgeon who had completed only 10. Our study has also shown this to be the case. Patients who underwent a RP between 2007 and 2009 had a lower PSM rate, compared to those in 2005–2006 (OR 0.312 vs OR 0.417). This was also true of patients undergoing surgery in 2005–2006 compared to those in 2003–2004 (OR 0.417 vs OR 1). The British Association of Urological Surgeons (BAUS) recently published surgeon reported data regarding transfusion, length of stay and complication rates across the UK but positive margin rates were not published [22]. The current study includes margin data and shows a clear link between surgeon experience and positive margin rates T2 disease. The surgeon in our study has had extensive training for the laparoscopic procedure. He undertook some assisting as a trainee and did part procedure cases as a primary surgeon. In addition he underwent 7 months of fellowship training in Institute Montsouris, Paris, which involved assisting, and gaining dry and wet laboratory training.

Our study only included T2 disease and therefore looked at the impact of surgical stage on PSMs on a finer basis. We showed a significant association between T2 PSMs and surgical stage between subclasses in T2 disease (T2 a, b and c). Increasing tumour volume makes incision of the prostate more likely in T2 disease.

Lower prostatic weight has also been shown to be an independent predictor of IPI due to the surgical difficulty involved in removing a small gland. [9, 23–25]. Marchetti et al. (2011) [25] found that predicted probability of a PSM was 22 % for patients with <25 g prostates, which decreased to just 1 % for those with >150 g prostates. Our study also showed lower prostatic weight (20–59 g) to be a predictor of T2 PSMs in the univariate analysis. In multivariate analysis, prostates >100 g had highest PSM rates. Patients with large prostates constituted a small number of more difficult cases and hence resulted in higher positive margin rates. Higher blood loss was associated with multifocal PSMs (*P* = 0.006). Higher blood loss is a surrogate for surgical difficulty and hence makes surgical incision of the prostate more likely.

PSMs have also been associated with nerve-sparing status. Due to the close approximation of the NVBs to the posterolateral prostate gland, this is the most common site for iatrogenic IPI [4, 5, 9, 10, 26]. Here, NVB status was not associated with margin location in the multivariable analysis, contradicting the literature [27]. Patients who did not undergo NVB sparing procedures had higher incidence of PSMs than those with nerve-sparing procedures. Non-nerve-sparing surgery was more likely in high-volume T2 disease. High-volume disease in the non-nerve spare group makes prostatic incision more likely (apex and base) and can explain why higher PSM rates were seen in this group of patients. A limit to our study was that information on the grade of nerve spare (partial, inter-fascial, intra-fascial) was not available as this may have an impact on the risk of PSMs.

IPI refers to iatrogenic incision into the prostate that contains cancer. There is great variability in the rates of IPI reported by different studies. For example, several studies have found incidence of IPI to be 1.8–2.8 % [9, 11, 28]. However, some studies have reported rates of IPI as high as 20 % [29]. Indeed, in our study, 30 patients (16.4 %) presented with PSMs in T2 disease (IPI). One possible explanation is difficulty in pathologic interpretation of surgical margin status, as has been highlighted by a number of studies [9–11]. There is a recognised danger for over-calling PSMs because of difficulty in differentiating between extraprostatic extension (EPE) and IPI [10]. Despite recently published guidelines on standardised handling of RP specimens by the International Society of Urological Pathology (ISUP) (2009), there is still a lack of consistency in the reporting of surgical margins [30].

A study by van der Kwast et al. [31]. highlights the inter-observer variation in the reporting of positive margin status. External review of pathology reports was carried out for patients in the European Organisation for Research and Treatment of Cancer trial (EORTC). There was only a 57.5 % agreement for extra-prostatic extension, and 69.4 % concordance for surgical margin status. A total of 24.9 % of the cases that were called positive at the local hospital were subsequently called negative on review in the study. More recent evidence has contradicted these findings, showing agreement in over 87 % of cases regarding margin status [32].

This evidence supports the fact that pathologists may interpret PSMs differently and calls into question the objectivity of surgical margin status as a quality measure. However, in our study, we found no such correlation between the reporting pathologist and margin status in both univariate and multivariate analysis. Therefore, it seems unlikely that the reporting pathologist has an impact on T2 PSM rates reported by different surgeons. It is more likely that any differences between T2 PSM rates reported by different surgeons are down to surgical experience, and tumour characteristics, not external bias.

One of the particular strengths of this study was the use of data from a single surgeon. This removes the heterogeneity of surgeon skills and allows valid comparisons of PSM rate over time. Only 30 PSMs were present in our study and therefore reduced the statistical power of our analysis. However, our PSM rate was comparable with other studies of a similar nature, for example Kumano et al. (2009) [29], suggesting that the population was representative. Another limitation of the current study was that data were missing for many of the patients in our database. This was particularly the case for nerve-sparing status. In our study, 48 patients (26.2 %) were missing data on nerve-sparing status. Of these 6 had a PSM at surgery, which represents 20 % of the PSMs in our population. This may be one reason why our study contradicted the majority of the literature with regard to the association of NVB resection and PSMs. Time to surgery was not evaluated in our cohort and would be interesting to study in the future, as waiting time may lead to tumour growth and therefore higher risk of PSM than is predicted by the clinical variables.

## Conclusion

In the absence of good patient reported outcomes, T2 positive margin rates are increasingly being used a quality outcome measure of surgeon experience. Our study has shown that T2 positive margin rates are not influenced by external biases such as the reporting pathologist but are affected by tumour characteristics and surgeon skill alone and can therefore be considered as an objective measure of surgeon skill.
